# Feasibility of tumor detection with a transmission‐based microwave imaging system

**DOI:** 10.1002/mp.18080

**Published:** 2025-09-12

**Authors:** Pedram Mojabi, Roger Y. Tsang, Bobbie Docktor, Danielle Deutscher, Anita Garland, Md Mahsin, Jeremie Bourqui, Elise Fear

**Affiliations:** ^1^ Electrical and Software Engineering University of Calgary Calgary Alberta Canada; ^2^ Department of Medical Oncology University of Calgary Calgary Alberta Canada; ^3^ Department of Radiology University of Calgary Calgary Alberta Canada; ^4^ Precision Oncology Hub Arnie Charbonneau Cancer Institute Calgary Canada

**Keywords:** breast imaging, microwave imaging, tumor detection

## Abstract

**Background:**

Microwave imaging has been proposed for breast cancer detection, relying on differences between the microwave frequency properties of healthy and cancerous tissues. Specifically, localized increases in permittivity and/or conductivity may be identified in images of the breast created with microwave tomography. In radar‐based images, responses may be noted where property changes occur. Our team has developed a microwave imaging approach that creates maps of permittivity by analyzing the characteristics of signals transmitted through the tissues, scanning the breast in views similar to mammography.

**Purpose:**

This study aims to assess the feasibility of tumor detection with a transmission‐based microwave imaging system. Specifically, both breasts of a control group and a group of women with a cancer diagnosis are scanned in both cranial–caudal (CC) and medial–lateral oblique (MLO) orientations to facilitate comparison with mammography.

**Methods:**

The microwave scanner consists of planar transmit and receive arrays that are placed in contact with the breast. The arrays are placed horizontally to collect the CC view, then tilted to an angle of 45 degrees to collect the MLO view. Signals with frequency content from 0.1 to 8 GHz are transmitted through the tissue, and the characteristics of the detected signals are used to estimate the microwave frequency properties (permittivity). Estimates at each sensor pair are mapped to the imaging plane. The average microwave frequency properties are calculated for the breast region identified in each image. Images are also segmented using k‐means and thresholding to further explore tumor detection. Statistical analysis is applied, specifically analysis of variance (ANOVA) to determine differences between views and groups.

**Results:**

20 healthy participants and 14 cancer patients are scanned. Right and left breasts are compared for each patient. Consistency is noted when comparing the CC scans of the breasts of healthy participants; similar observations are made for the MLO scans. Similarity between CC and MLO views of the same breast is also noted and confirmed via ANOVA testing. For patients with confirmed cancer, greater differences are noted between the breasts in at least one view when observing images and average properties. The ratio of the average permittivity of the cancerous breast to the contralateral breast is significantly different than the ratio observed in healthy volunteers when considering CC views. Greater ratios are also observed for patients with higher breast densities.

**Conclusion:**

This study demonstrates the potential for a transmission‐based microwave scanner to detect tumors.

## 1 INTRODUCTION

Breast cancer is an important issue for women, and imaging is a key component in early detection, diagnosis, staging and follow‐up. Breast cancer screening programs are designed to promote early detection of breast cancer and involve regular mammograms.^1^, ^2^ Several additional approaches to breast imaging are well established clinically but have acknowledged limitations.^3^ Diagnostic and contrast‐enhanced mammography (CEM) are used to investigate clinical concerns (e.g., pain, palpable lesions). CEM is also used for pre‐operative cancer staging and is being explored for treatment monitoring. However, mammography involves exposure to x‐rays, which limits frequency of scans, and also exhibits reduced sensitivity in dense tissues.^4^ Ultrasound may be added to screening for women with dense breasts and is used to further evaluate suspicious lesions, but diagnostic performance is highly user dependent.^5^ While automated breast ultrasound reduces acquisition time, interpreting images can be time‐consuming. MRI is used for women at high risk (e.g., BRCA gene carriers), to stage cancer, and for treatment monitoring when available.^6^ Other approaches, such as quantitative ultrasound, breast‐specific PET, and optical imaging have been proposed,^7^, ^8^, ^9^ but have not gained clinical traction.

Clinical use of breast imaging modalities depends on standards of care and a range of complex factors including availability and access to resources, leading to greater survival rates in developed regions than developing ones.^10^ Even within developed countries, participation in breast screening may vary; for example, within Canada, participation in screening is impacted by socioeconomic status, location, and ethnocultural group.^11^ Access to imaging technologies during breast cancer treatment may also be impacted by these factors. For example, MRI is recognized as the optimal technology for monitoring disease response during neoadjuvant chemotherapy, but requires scans prior to and during therapy.^12^ Accessing this technology in a timely manner may be difficult for patients who do not live in urban centers. These challenges have generated interest in the development of new breast imaging technologies that are suited to regular scanning and easily transported to and operated in rural and remote locations. A promising alternative technology, microwave imaging (MWI), has attracted growing interest since it is inexpensive, comfortable for patients, and does not require ionizing radiation.^13^

MWI is based on differences in MW frequency (MWF) properties of tissues (permittivity and conductivity), which relate to water content.^14^ These properties have been assessed in several studies using dielectric probe measurements of excised tissues and analyzed via comparison with tissue composition under the probe location obtained from pathology slides. In two studies, excised tissues from breast reductions (354 samples) and cancer surgeries (85 normal, 60 cancer, 10 benign samples) were measured over the frequency range of 0.5 to 20 GHz.^15^, ^16^ The results showed a wide range of properties for healthy tissues and differences on the order of 10% for tumors and predominantly glandular tissues. Another study of 102 samples from 35 cancer patients measured from 0.5 to 20 GHz^17^ also showed a range of properties, attributed to the fractions of adipose and stroma cells noted in pathology slides. When comparing samples from individual patients at 6 GHz, differences in properties of adipose and stroma tissues were noted, as well as increased properties when comparing stroma and tumors samples in most cases. Further analysis indicated that tumor samples included 10% to 80% stroma cells and the higher measured permittivities related to higher cancer cell densities. A study of 166 normal and 56 tumor samples measured from 0.5 to 50 GHz also showed a wide range of properties; comparison of average properties of high, medium and low density samples to tumor tissues showed differences across all densities (e.g., 12% to 20% in real permittivity and 22% –28% in imaginary permittivity compared to high density samples).^18^ Finally, a study examining 128 cancer, 224 normal and 157 benign samples showed different average properties of these tissue categories.^19^ Therefore, excised tissue measurements indicate permittivity and conductivity contrasts between healthy and cancerous tissues to enable tumor detection, albeit complicated by the range of properties of healthy tissues. Interpretation of these results is also complicated by the inherent heterogeneity of tissues in the sensing volume of the probe^20^ and the need for tissue handling procedures that conserve moisture.^18^

Several approaches to microwave imaging have been proposed to leverage the observed property contrasts between healthy and malignant tissues. While this idea was introduced in the 1940s,^21^ practical approaches to microwave breast imaging were initially developed and tested in work ranging from simulations^22^ to scans of patients^23^ and have been extended to systems used in larger scale studies of human subjects (reviewed in Porter and O'Loughlin^13^). The basic approach to imaging involves illuminating the breast with microwaves and detecting signals transmitted through or reflected from tissues. Transmitted signals are used to form images in different ways: radar‐based techniques that focus reflections,^24^, ^25^, ^26^, ^27^, ^28^ tomography methods utilizing model‐based reconstructions,^29^, ^30^, ^31^, ^32^, ^33^ scattered power mapping^34^, ^35^ and uniform plane wave approximations.^36^, ^37^ When considering imaging the intact breast with microwave tomography, increased permittivity and conductivity have been noted when comparing regions corresponding to known tumor locations with other regions in the same or contralateral breast.^38^ Radar‐based imaging studies have noted increased scattering in the breast containing the tumor. While tumor detection has been demonstrated in groups of patients with both approaches (as summarized in^13^), comparison to clinical images is complex because patient positioning is typically prone, differing from the views collected in mammography.

Our team has developed a planar microwave imaging transmission system (TMS). Maps of MWF properties are created by analyzing the characteristics of signals that have traveled through the breast tissues in comparison to a reference.^36^ TMS images are maps of the underlying MWF properties. The TMS collects scans of both breasts in minutes and comparison to mammography is facilitated because our microwave system positioning is similar to the dual‐plane views used in mammography. Our previous studies have focused on establishing reproducibility^39^, ^40^ and exploring links between MW scans and breast composition reported in mammograms.^41^ In this paper, we aim to assess the feasibility of detecting tumors with the TMS approach by comparing scans of both breasts for a group of patients and a cohort of healthy participants. Specifically, we present scans of both breasts of 14 patients with known lesions and 20 participants without known breast disease. In our previous work, only cranial–caudal (CC) views have been reported. Here, both cranial–caudal (CC) and medial–lateral oblique (MLO) views are collected and analyzed. These two views enhance comparison with mammography, as well as exploration of the potential to detect tumors, as lesions may only be evident on one of the two views. First, sample images for patients and participants are shown. The average properties of the images are compared as an initial exploration of detection, then more detailed approaches in which images are segmented via k‐means or thresholding are also tested. Finally, a one‐way analysis of variance (ANOVA) is used to evaluate differences between right and left breasts for healthy participants and patients, and a two‐way ANOVA is performed to compare the means of the two groups.

## 2 METHODS

### 2.1 Microwave imaging system

**FIGURE 1:**
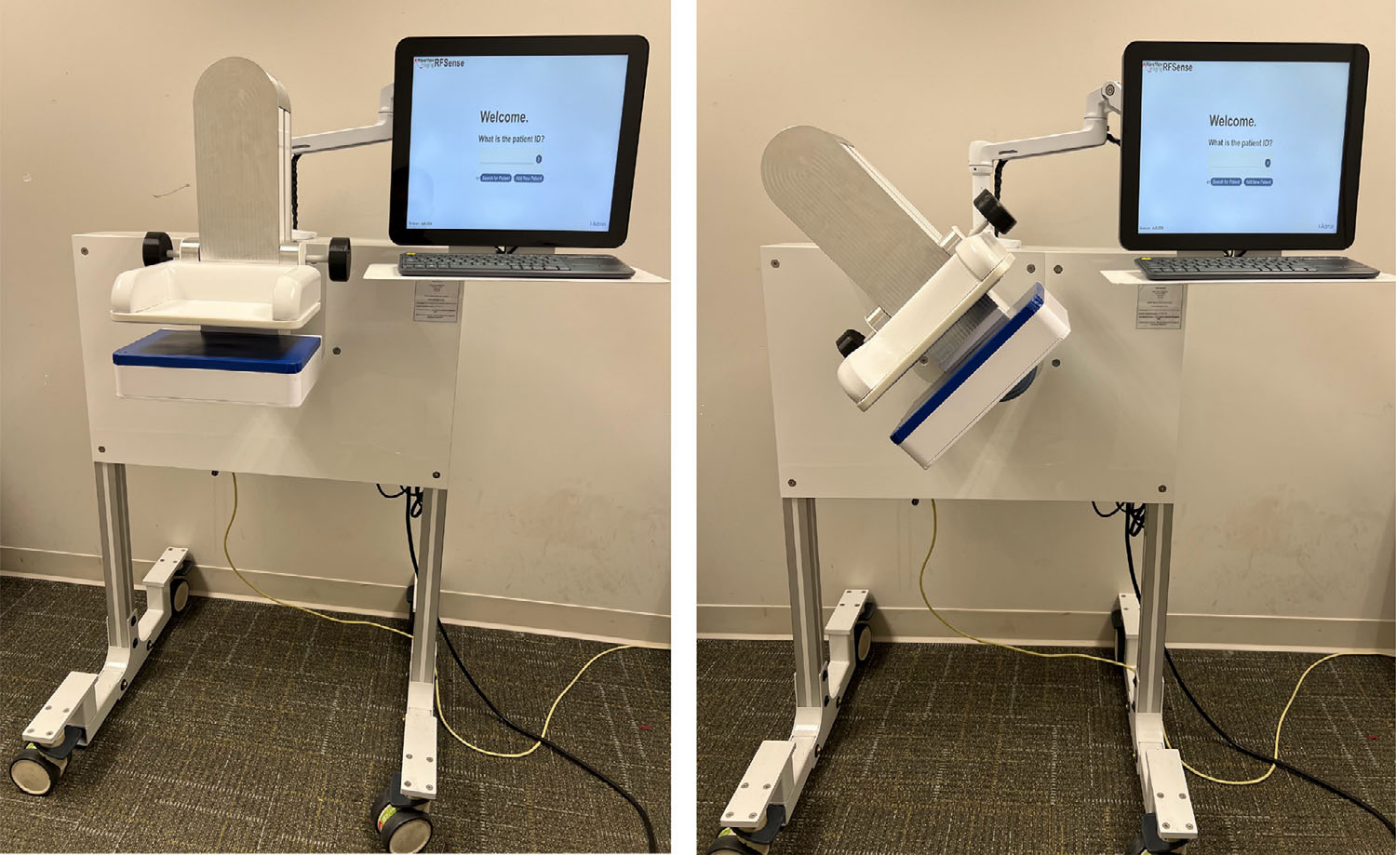
Microwave imaging transmission system for breast scanning: (left) system in position to collect craniocaudal (CC) scans; (right) system in mediolateral oblique (MLO) position.

The TMS, shown in Figure 1, consists of two high density arrays of antennas that operate over an ultrawide frequency band.^42^ The breast is positioned between the two plates and the top plate is lowered to contact the breast. Signals passing through the breast tissues are measured from 0.1 to 8 GHz and transformed to the time domain. The time of arrival of the signal is identified and compared to the time of arrival of a signal passing through the same volume of air; the difference is used to estimate the MWF properties of tissues between each sensor pair. These estimates are then mapped to a 2D plane to form the image.

As a similar microwave imaging system has been previously described,^42^ only a brief summary is provided here. When scanning, the woman sits in front of the system and her breast is placed on the lower plate. The upper plate is lowered to contact the tissue. This approach allows a range of breast sizes to be scanned. For the MLO view, the plates are rotated by 45 degrees, as shown in Figure 1. The top plate, serving as the transmitter, contains 247 low‐profile patch antennas, while the bottom plate, acting as the receiver, consists of 240 receiving antennas. These antennas operate over a wide frequency range from 0.1 to 8 GHz. This planar system features a large imaging plate capable of accommodating breasts of various sizes. The maximum imaging area is approximately 21 cm×16 cm. This increased number of antennas, particularly in the transmitting array, is the key difference between this system and the previous implementation.^36^

When a scan is collected, signals are transmitted through each antenna in the upper array in turn. After traveling through the tissue, the signal is detected on the opposite plate at a set of antennas in proximity to the transmitter. A vector network analyzer (C2409, Copper Mountain Technologies, Indianapolis, USA) in conjunction with a custom switching matrix is used to collect measurements. The total number of paths measured is more than 2000, allowing for the entire breast to be scanned. The measurements required for one image are acquired in less than 1 min.

While the measurements are collected in the frequency domain, the estimation of MWF properties requires time‐domain signals. First, the signals are transformed to time domain with an inverse chirp Z‐transform. The peak of the resulting signal is compared to the peak of a signal traveling through a reference material of the same thickness. The difference in arrival times is used to estimate the relative permittivity ($\varepsilon_{r}$) of the tissues in the sensing volume of the transmitter/receiver pair.^36^ Further references to permittivity (or average permittivity) refer to the relative permittivity. To form a 2D image, the permittivity estimate at each transmitter/receiver pair is mapped to a footprint on the plane located mid‐way between the arrays.

### 2.2 Participants

For this paper, scans from participants enrolled in three separate studies are analyzed. Studies 1 and 2 involve healthy participants. Participants (age 18 and over) were recruited from the Calgary community via the Participate in Research website and contact with the research team. None of these participants had a recent cancer diagnosis or were undergoing breast cancer treatment at the time of enrollment. Participants in the second study had a previous mammogram. The third study included women diagnosed with breast cancer who were invited to participate by their medical oncologist. Participants in this study received a diagnosis of early‐stage or locally advanced breast cancer and were scheduled to receive neoadjuvant chemotherapy as their initial treatment prior to surgery. Inclusion criteria included tumor stage, specifically T1C (tumors of 1 cm or greater), T2, and T3 with any lymph node status (N any) and no metastasis (M0). For all except one patient (as described below), the scans reported in this paper were collected prior to starting neoadjuvant chemotherapy treatment. As this was a pilot study, sample size calculations were not performed. All participants enrolled in the studies provided written informed consent. The studies were approved by the Health Research Ethics Board of Alberta ‐ Cancer Committee (HREBA.CC‐21‐0082, 22‐0031, and 22‐0333).

Previous mammograms (images and reports) were accessed when available for the control group and for all patients. The imaging reports included an assessment of breast density (breast volume, percentage density, and density category of A, B, C or D as defined by the Breast Imaging and Reporting Data System, American College of Radiology). Density categories A, B, C, and D correspond to fatty breasts, scattered areas of fibroglandular tissue, heterogeneously dense breasts, and extremely dense breasts, respectively.^43^ Thus, from A to D, breast density increases with a greater proportion of glandular tissue. These quantities were available in the reports on the mammograms and were calculated with an automated breast density assessment tool (Volpara Health, Wellington, New Zealand). For patients, the pre‐treatment mammograms or ultrasound scans and biopsy information were accessed to obtain information on tumor size and type.

During each session, the left and right breasts of the participant were imaged in the CC and MLO orientations. A registered nurse operated the scanner. The volunteer positioned her breast in the scanner on the lower plate. The nurse decreased the separation distance until good contact was made between the upper plate and the breast. A measurement was collected, then the separation distance was decreased by a target of 5 mm and a second set of measurements was collected. Therefore, images were collected at two separation distances per scan, referred to as D1 and D2. In this paper, the data from the smaller separation was used to create permittivity image of the breast. The CC views were collected first, then the scanner plates were tilted by 45 degrees (in each direction) to collect the MLO views.

### 2.3 Comparison to clinical data

The images of both breasts were examined, and quantitative analysis was applied. After the image was formed, k‐means clustering was used to identify the breast area. The average value of the permittivity in the region representing the breast was calculated for all 4 scans. The properties were compared by taking the difference between the two breasts and normalizing to the average value of the images collected in the same view. We note that previous work^44^ suggested that differences of 10% or less in the average properties of the healthy right and left breasts were typical. To better interpret the images and average properties, the separation distance between the plates and the area of the breast region were included. Increases in average properties of 5% were typically noted with decreases in separation distance of 5 mm.^44^ In the cohort of participants examined, the differences between right and left breasts that were outside of the indicated ranges often corresponded to smaller breasts; this was also noted for changes in separation distance. Increased coefficient of variation was observed for the average permittivity when considering repeated scans of smaller breasts, suggesting challenges in positioning smaller breasts in the scanner.^44^

Finally, the images were analyzed in greater detail, First, k‐means was used to segment the breast into two regions representing tissues with lower (fat) and higher (glandular tissues, tumors) values. The K‐means method groups data into k clusters based on value similarity. In this study, we use two clusters to segment the image into two regions: one representing lower permittivity, corresponding to fat tissue, and the other representing higher permittivity, corresponding to glandular tissue. The built‐in K‐means function in MATLAB is used for this segmentation. Second, thresholds were applied to segment the images into regions corresponding to different tissues. Specifically, the images are segmented into 4 regions using a consistent set of values for all participants. The range of permittivity for each region is chosen as: 0<R1≤8, 8<R2≤13, 13<R3≤18 and R4>18. These ranges were identified through scans of participants in previous studies.^44^ Region 1 denoted by R1 corresponds to the lowest properties (fatty tissue), while region 4 denoted by R4 corresponds to the highest properties. The area of each region relative to the breast area was calculated.

### 2.4 Statistical analysis

In this study, a one‐way analysis of variance (ANOVA) was performed in the R programming language to evaluate the statistical significance of the differences between the breasts. Both CC and MLO views were considered in the analysis of average permittivity, separation distance, and breast area. This analysis was conducted separately for healthy participants and those with cancer. Additionally, a two‐way ANOVA was performed that included both healthy participants and cancer patients. To compare the means of the two groups, two‐sample *t*‐tests were used. *p*‐values were calculated as two‐tailed when appropriate, with a significance threshold set at less than 0.05. Finally, a one‐way ANOVA was used to analyze the ratio between the properties of breasts for both healthy participants and patients.

## 3 RESULTS

A summary of the healthy participants is provided, including sample images, average properties and image segmentation results. This is followed by a similar summary for cancer patients. As these are small cohorts, additional descriptions are provided to aid in interpreting results. The results for all participants and corresponding statistical analysis are provided.

### 3.1 Healthy participants

Table 1 provides a summary of the controls, specifically age and breast density (if known). Not all participants in study 1 had a previous mammogram.

**TABLE 1:** Summary of available data for 20 healthy participants.

ID	Age	Breast Density
1	36	
2	45	
3	37	
4	40	
5	75	D
6	45	C
7	46	B
8	23	
9	47	B
10	40	C
11	31	
12	68	A
13	65	B
14	40	C
15	63	C
16	39	D
17	62	C
18	68	B
19	44	C
20	58	B

#### 3.1.1 Sample images

**FIGURE 2:**
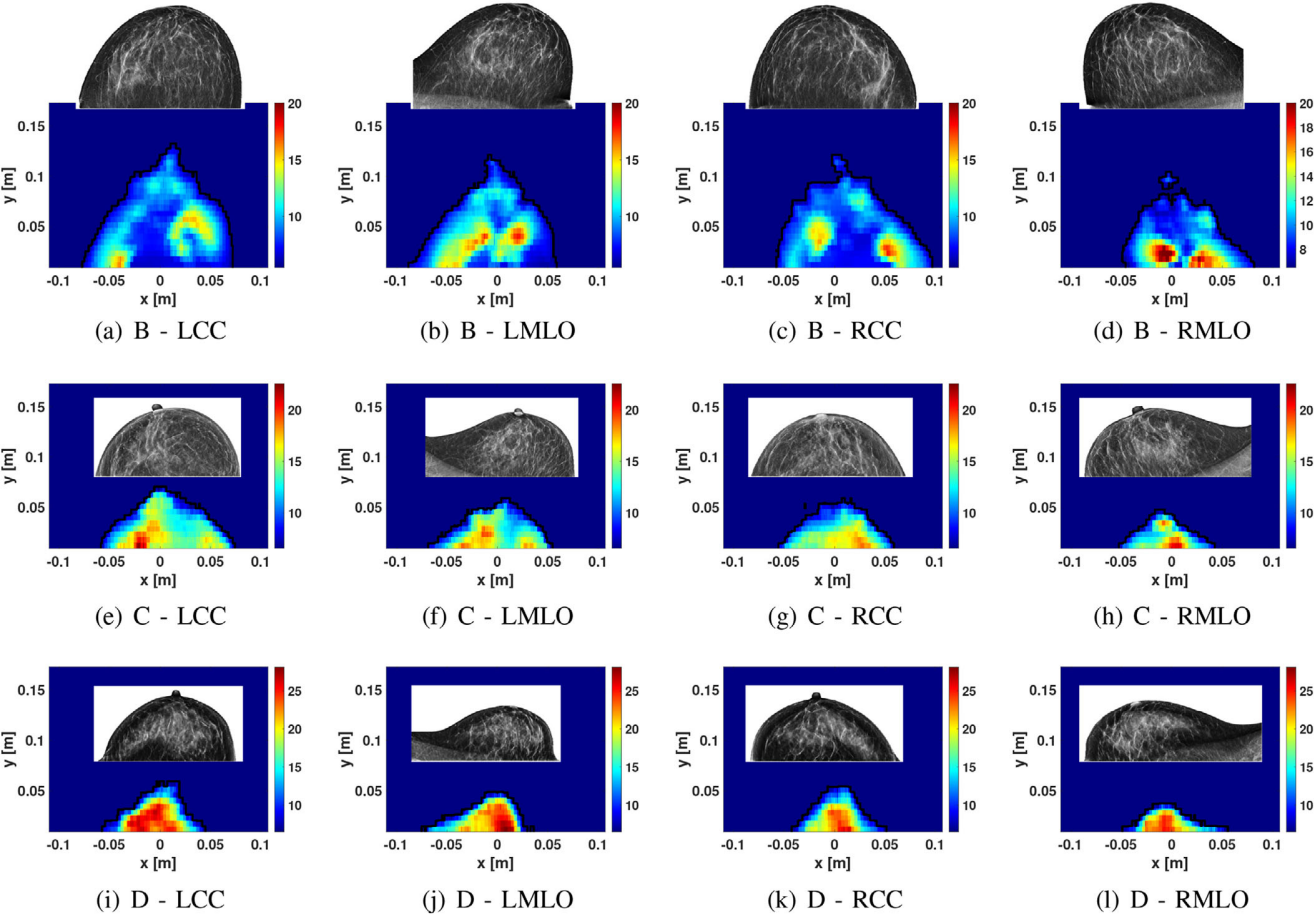
Sample images of healthy participants. Each row corresponds to an individual participant and each of the participants has a different breast density from B to D. The first, second, third, and fourth columns correspond to the LCC, LMLO, RCC, and RMLO images, respectively. The corresponding mammogram is displayed with the microwave reconstruction. The first, second, and third rows correspond to volunteers with IDs 7, 15, and 16, as listed in Table 1.

Figure 2 presents sample images of healthy participants with different breast compositions. Both the CC and MLO views are included, along with the corresponding mammograms. For each volunteer, the same colorbar is used to display the images. In the first row, the breast density of the participant is scattered (category B indicated on the report on the mammogram). Similarity in the reconstructed properties is noted in the CC and MLO views of the same breast, as well as between the breasts. Fatty tissues have lower properties and glandular tissues have higher properties; the regions of increased properties likely correspond to glandular tissues. In the second row, the participant had breast density of C. Compared to the first row, a greater portion of the breast exhibits higher properties. The third participant has breast density of D. Increasing properties are noted with increasing breast densities, while similarity between properties reconstructed in both views of the breast are also observed. It should be noted that the maximum of the colorbar also increases from breast density B to D.

#### 3.1.2 Average properties

**FIGURE 3:**
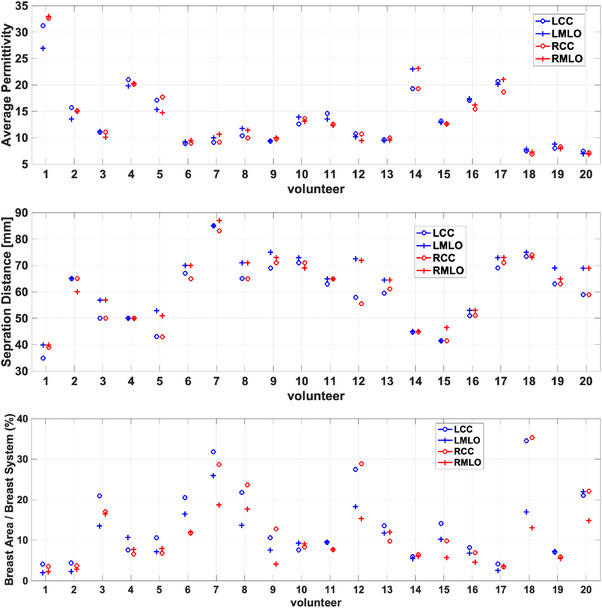
Combined data of the healthy participants. The first, second, and third rows correspond to the average permittivity, separation distance, and ratio of breast area to the system, respectively. For each participant, the blue color corresponds to the left breast and the red color corresponds to the right breast. The circle represents the CC position and the plus sign represents the MLO position.

The average properties of each image are calculated and summarized in Figure 3. This figure illustrates the similarity in properties for the CC views and the MLO views; for many participants, all four views exhibit similar average properties. Participant 1 has a lower average for the LMLO view. To assist in interpretation, Figure 3 also summarizes the separation distances between the plates and the area of breast region in the image relative to the area of the system. For participant 1, the separation distance for the LCC view is 5 mm smaller than the RCC view. In previous work, decreases in separation of 5 mm resulted in property increases of approximately 5%.^44^ We also note that the breast area for participant 1 is less than 5% of the image; the areas of the MLO views are less than the CC. Positioning smaller breasts in the scanner is challenging and average properties are calculated over a very small region, which can result in greater differences between breasts. The breast area of participant 2 is also small, and a decreased average for the LMLO view is also noted. Participant 3 has similar properties for all views; while the separation distances for the MLO views and CC views are similar, the differences between the MLO and CC views are more than 5 mm. This participant has lower properties and a larger breast area, which is likely why less impact of positioning is noted. Similar observations are made for participants 12 and 20. Participant 5 exhibits differences in separation between CC and MLO; the CC properties are correspondingly higher. In spite of differences in separation between CC and MLO for participants 6 to 9, the average properties of the images are similar. In general, differences in separation distance between CC and MLO do not consistently result in differences in properties. This is likely related to the difference in position of the breast.

To assess the variability among healthy subjects, a one‐way ANOVA was conducted to compare results obtained for the LCC, RCC, LMLO and RMLO views. The analysis revealed no statistically significant differences in the average permittivity between the CC and MLO views of the left and right breasts (*F*‐value = 0.01, *p*‐value > 0.9). Similarly, no notable differences were observed for the separation distances between the plates (*F*‐value = 0.63, *p*‐value = 0.6) or the ratio of breast area to the imaging system (*F*‐value = 1.5, *p*‐value = 0.2) when comparing the results obtained for all 4 views.

#### 3.1.3 Segmentation

**FIGURE 4:**
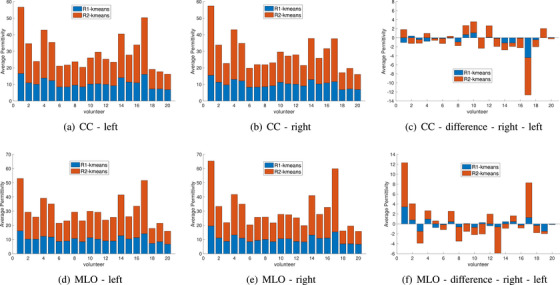
Combined data for k‐means segmentation of the images of 20 healthy participants. Average permittivity of two regions identified by k‐means segmentation is calculated for left and right breasts in CC and MLO positions. The two average values are stacked, so the overall height of each bar is the sum of the two values.

Next, the images are segmented using a k‐means approach and thresholding. Figure 4 summarizes the average properties of the two regions obtained by k‐means segmentation for all 4 views. Specifically, k‐means is used to identify regions dominated by lower and higher permittivity values. In Figure 4, the average properties of the two regions are stacked, so the total height of each bar represents the sum of the average values. Similar patterns are noted for each participant when comparing the two CC or the two MLO views; there are also similarities between the CC and MLO of each breast. The differences in the average permittivity between these two regions for the left and right breasts in the CC and MLO positions are shown in the last column of Figure 4. It can be observed that this difference is particularly high for participant 17 in the CC position and for participants 1 and 17 in the MLO position. It should be noted that as shown in the last row of Figure 3, volunteers 1, 2, and 17 have very small breast areas (the ratio of area breast to area system is less than 5%). Overall, this demonstrates the consistency of the properties imaged for all views. The differences between the average values also tends to show greater differences for the MLO view.

**FIGURE 5:**
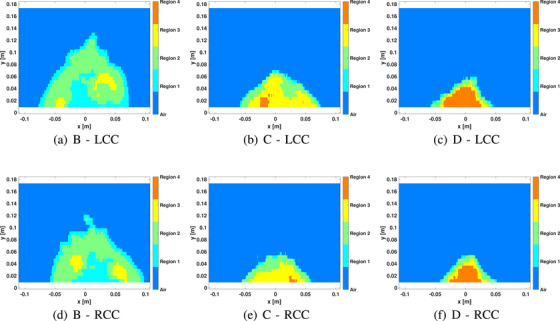
Sample region‐based images of three healthy participants with different breast density categories. A threshold is used to define each region of the breast. Each column corresponds to a specific participant with different breast density categories from B to D (volunteers with IDs 7, 15, and 16 in Table 1). The first and second rows correspond to the left and right breasts, respectively, in the CC position. The percentage of each region for the images in the first row are as follows. For the B density, R1≃34.85%,R2≃57.80%,R3≃7.35%,R4=0. For the C density, R1≃8.68%,R2≃33.62%,R3≃52.76%,R4=4.94%. For the D density, R1≃10.41%,R2≃24.23%,R3≃15.48%,R4=49.88%.

**FIGURE 6:**
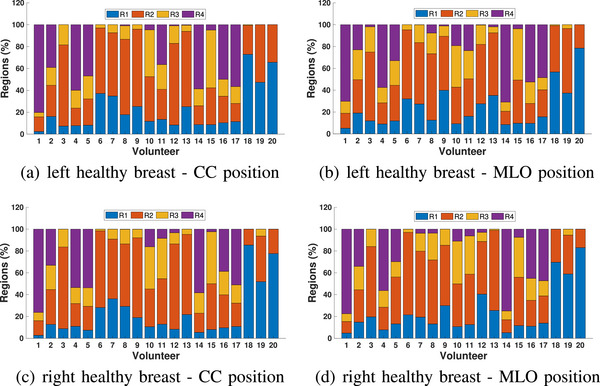
Combined data of the threshold‐based segmentation of images of 20 healthy participants. The area of each region relative to the area of the breast is calculated for left and right breasts in CC and MLO positions based on the threshold values.

To illustrate the threshold approach, the permittivity images of the healthy volunteers shown in Figure 2 for the CC position are segmented into four regions based on threshold, as shown in Figure 5. The symmetry of the left and right breasts of these healthy volunteers is clearer using these images. For these participants, region 2 is dominant for density B, region 3 for density C and region 4 for density D. Results for all participants are summarized in Figure 6. This more detailed analysis demonstrates consistent patterns for the CC views of both breasts, as well as the MLO views. There are differences between CC and MLO, however this is expected due to differences in positioning.

Overall, the results in this section demonstrate the similarity between the CC scans and the MLO scans of both breasts. All scans of the same participant also exhibit similarity. This is observed across average properties, as well as images segmented with k‐means and threshold‐based approaches. This similarity provides a basis for further exploration of tumor detection.

### 3.2 Participants with cancer

Table 2 provides a summary of the patient age, breast density, tumor size and type. The tumor size shown in the fifth column of Table 2 was obtained from the imaging study closest to the start of treatment, which is most often an ultrasound. The number of days between biopsy and the first scan using our microwave system is approximately 50 days, with a minimum of 26 days and a maximum of 80 days. This time frame is generally sufficient for the transient effects of biopsy trauma to subside, thereby minimizing the likelihood of significant residual tissue perturbation affecting the microwave measurements. For example, a case study using diffuse optical spectroscopy to monitor breast tissue after core biopsy found that tissue optical properties returned to baseline within minimum of 14 days.^45^

**TABLE 2:** Summary of data for 14 patients with breast cancer. IDC indicates invasive ductal carcinoma and DCIS indicates ductal carcinoma in situ.

ID	Age	Breast Density	Tumor Location	Tumor size (cm)	Tumor Type
1	42	B	R	2.1×1.8×1.9	IDC (ER‐, PR‐, HER2‐)
2	46	D	L	2.6×1.6×1.5	IDC (ER‐, PR‐, HER2‐)
3	68	B	R	2.2×1.2×2.6	IDC (ER‐, PR‐, HER2‐)
4	54	C	R	2.7×2.3×1.5	IDC (ER+, PR‐, HER2+)
6	37	C	L	2.2×1.2×1.5	IDC (ER+, PR+, HER2+)
7	61	A	L	2.2×1.4×1.8	IDC (ER+, PR+, HER2+) + DCIS
8	38	D	R	5.2×4.2×6.0	IDC (ER‐, PR‐, HER2‐)
9	49	C	L	2.0×1.8×2.5	IDC (ER+, PR‐, HER2‐)
10	58	B	R	4.9×2.5×5.7	IDC (ER+, PR+, HER2‐)
11	32	B	R	3 lesions	IDC (ER+, PR‐, HER2+)
12	39	B	L	10×11×10	IDC (ER+, PR‐, HER2+) + DCIS
13	56	C	L	2.8×1.7×2.3	IDC (ER‐, PR‐, HER2+)
14	63	B	L	1.6×1.4×3.0	IDC (ER+, PR+, HER2‐)
15	53	C	R	3.4×1.8×2.1	IDC (ER‐, PR‐, HER2+)

Patient 7 had extensive calcifications suggestive of DCIS in addition to IDC. Patient 11 had 3 lesions (one at 11 o'clock with size 1.8 x 1.0 x 1.8 cm; a second in the axillary tail at 10 o'clock (2.0 x. 1.5 x 2.0 cm) and a third retroarelolar lesion (0.5 x 0.3 x 0.4 cm). Patient 12 had DCIS with extensive calcifications in the entire lower half of the breast. Finally, the lesion in patient 15 was located adjacent to the chest wall. For patient 8, the first scan (baseline) of the breast with the tumor had a very large separation distance between the two plates of the microwave system. This could be due to inflammation, which leads to discomfort when the plates of the microwave scanner are in contact with the breast. The separation distances for the first and second scans is about 87 and 60 mm, respectively. Therefore, for this volunteer only, we use the data of the cancerous breast after the first session of chemotherapy (second scan) rather than the baseline scan.

#### 3.2.1 Sample images

**FIGURE 7:**
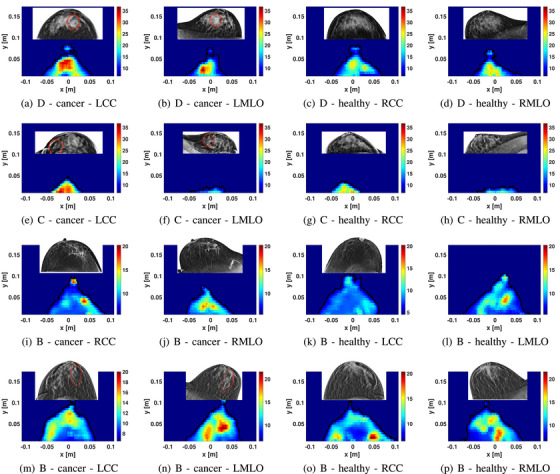
Sample images of patients with a cancer diagnosis. Each row corresponds to a particular patient (patients 2, 6, 10, and 12). The first two columns show CC and MLO views of the breast with the tumor, and the last two columns show the contralateral breast. The subfigures (e) and (g) were taken from^46^ and rearranged, ©2024 IEEE.

Figure 7 provides images for 4 patients with single lesions. For each volunteer, the same colorbar is used to display all images. The available corresponding mammograms are display with the MW images. The location of the tumor in the mammogram is indicated by a red circle or annotations.

The first row, patients 2 with breast density D, shows clear differences between the breasts in both views. As can be seen, the maximum permittivity for the left breast in both the CC and MLO positions is much higher than that of the healthy right breast. In the second row, differences are clearly evident in the CC views for patient 6, who has breast density of C. The high permittivity value in the bottom left of the LCC image corresponds to the expected tumor position, marked with a red circle in the LCC mammogram. The MLO view illustrates challenges with positioning smaller breasts. Together with the result for patient 2, these intriguing results suggest potential for detection in dense tissues.

The third row shows images of patient 10, with a localized increase noted in the CC view of the right breast. Increased properties are noted in the MLO view, along with greater values in two localized areas. In the corresponding mammograms, the responses are similarly located and evident in both views. The position of the tumor for this volunteer is shown with a white dotted line, and the mammogram for the MLO view of the healthy breast is not available.

Patient 12 is shown in the last row of Figure 7. This patient had breast density of B. Increases are noted in both views of the left breast a localized region of increased properties in the MLO review. This location is similar to the tumor location in the mammogram, as indicated with a red circle. A larger region of tissue is impacted compared to patient 6 and the responses in the microwave images are evident over a greater region.

**FIGURE 8:**
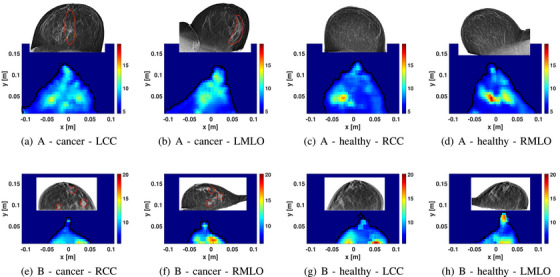
Sample images of patients with multiple lesions. The first and second rows show the permittivity reconstruction for patient 7 with breast density A and patient 11 with breast density B, respectively.

Figure 8 summarizes results for two patients with multiple lesions. Patient 7 was diagnosed with IDC and a region of DCIS was also present. Patient 11 had 3 lesions; one lesion was located in the axillary tail of the breast, which is challenging to image as this portion of the breast may not be present in the scanner without manipulation of the breast tissue performed with mammography.

#### 3.2.2 Average properties

**FIGURE 9:**
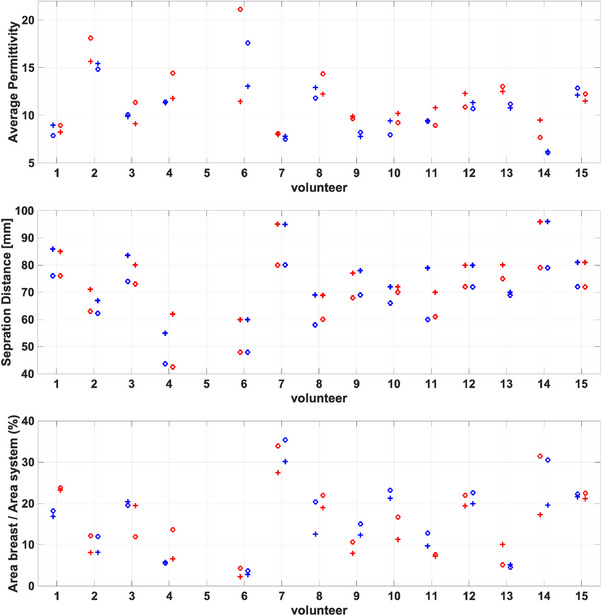
Combined data for the patient cohort. The first, second, and third rows correspond to the average permittivity, separation distance, and ratio of breast area to the system, respectively. For each patient, the left values correspond to the left breast and the right values correspond to the right breast. The blue color corresponds to the healthy breast and the red color corresponds to the cancerous breast. The circle represents the CC position and the plus sign represents the MLO position.

Figure 9 summarizes the average properties for CC and MLO views of the breasts with and without cancer. The red labels indicate the breast with the tumor, while the blue indicate the contralateral breast. For all but two patients, at least one view of the breast with cancer exhibits higher properties than the breast without cancer than would be expected based on the scans of healthy participants. For patient 15, the tumor was located adjacent to the chest wall and was likely not in the scanned volume of the breast. Patient 7 shows localized regions of greater properties in the breast without the tumor; while the breast with the tumor shows larger regions of elevated properties, the overall averages for all breasts are similar.

There are several additional observations for specific participants. Patient 11 has a greater separation distance in the MLO view for the breast without cancer, which may be responsible for lower properties observed when compared to the breast with the cancer. For patient 6, the average permittivity of the cancerous breast is much higher than that of the healthy breast in the CC position. However, we do not observe the same pattern in the MLO position. This participant had a smaller breast size and a small breast region is noted in the permittivity reconstruction for this volunteer in the second row of Figure 7.

A one‐way ANOVA was applied to analyze the results obtained for LCC, RCC, LMLO and RMLO views. This showed statistically significant differences in the separation distance between CC and MLO views (*F*‐value = 3.8, *p*‐value = 0.015). However, the average permittivity (*F*‐value = 0.25, *p*‐value = 0.9) and the ratio of breast area to the system (*F*‐value = 0.48, *p*‐value = 0.7) did not show statistically significant differences. The range of properties observed for the right and left breasts likely obscured differences between the breasts with and without cancer. This is further analyzed in the next section.

#### 3.2.3 Segmentation

**FIGURE 10:**
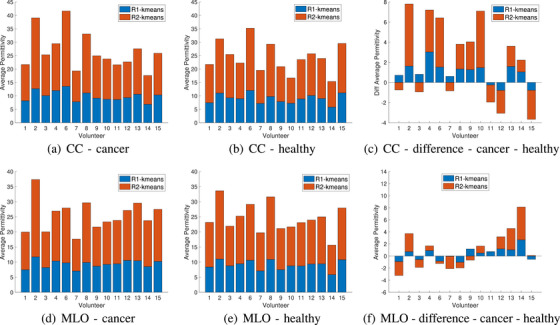
Combined data of the 14 patients for k‐means segmentation. Average permittivity of two regions is plotted for breasts in CC and MLO positions. The two average values are stacked

Figure 10 summarizes the average properties of the two regions resulting after segmentation. Again, the total height of each bar represents the sum of the average properties of the two regions. Patients 2, 4, 6, 8, 9, 10, and 13 exhibit increases in the CC view. Patients 2, 12, 13, and 14 exhibit increases in the MLO view. When compared with the healthy participants, greater differences between the two breasts are noted in the CC view. It should also be noted that for cancer patients, the ratio of breast area to system area is higher than 5%, except for patient 6, as shown in the last row of Figure 9. Furthermore, when comparing the changes in average permittivity between these two regions for cancer and healthy participants with an area ratio greater than 5% in the CC positions, a greater difference is observed for cancer patients compared to healthy volunteers.

**FIGURE 11:**
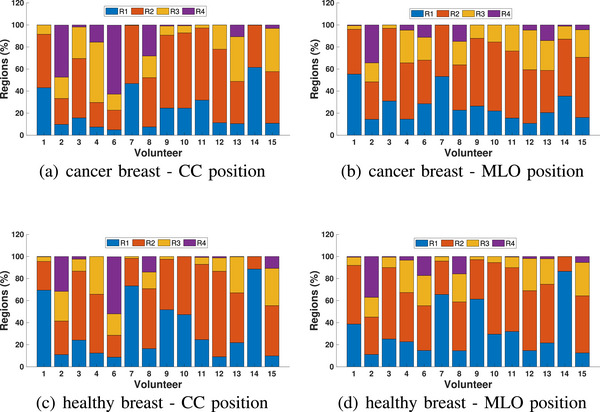
Combined data of 14 patients for the region‐based segmentation. The threshold values define four different regions, and the areas of these regions are compared to the area of the breast. Results are shown for CC and MLO views.

Figure 11 shows the results of the threshold segmentation. Comparing the breasts with and without cancer, the majority of patients exhibit increased relative areas of regions with higher properties in the CC view (i.e., regions 3 and 4). Patients 9 to 14 exhibit increases in these regions in the MLO view when comparing the two breasts. This demonstrates the utility of collecting two views of the breast to increase confidence in tumor detection, similar to mammography.

### 3.3 Analysis

**FIGURE 12:**
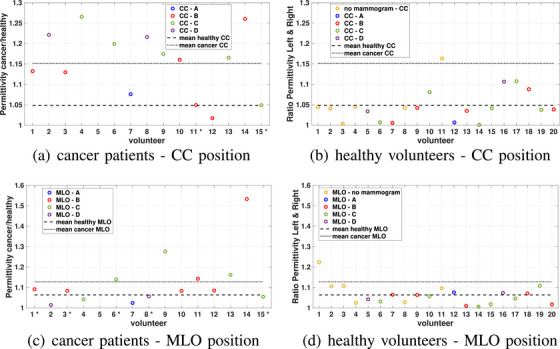
The ratio of the average permittivity of the left vs. right breast. The first column corresponds to patients with cancer, and the second column corresponds to healthy participants. The first row is based on the CC position, and the second row is based on the MLO position.

Figure 12 compares the average properties of the right and left breasts via the ratio between the higher and lower average property values. When considering the CC view, the average ratio for healthy participants is 1.05, while for cancer patients this ratio is 1.15. For the MLO view, the averages are similar for both groups. For most patients in this study, it appears that the tumors are more evident in the CC view, which is likely because breast positioning is easier in the horizontal CC view. In the CC position for patients 11 and 15, the average permittivity of the healthy breast is slightly higher than that of the cancerous breast, as indicated by a star symbol in the x‐axis label of the plot. For patients 9 and 14, the ratio for the MLO view is much greater than the average. The ratio is also higher for the CC view for these patients. Patients 11 and 12 show ratios in the MLO view that are greater than the average for the healthy participants; these patients were at or below this average in the CC view. For these patients, the tumor presents a clearer response in the MLO mammogram. For patient 15, the tumor was likely not in the imaging volume. The breast is placed on the lower plate and the upper plate is lowered to contact the tissue, so the regions of the breast near the chest wall and axilla are not scanned. Detecting tumors in these regions would be enabled through mammographic positioning techniques.

The breast density categories are also shown in Figure 12 with different colors. For the healthy participants, mammograms and breast density information are available for 14 of 20 volunteers. As can be seen in Figure 12a, the ratio of the average permittivities for patients with dense breasts (category C) and extremely dense breasts (category D) are greater than the mean ratio. Only patient 15, who has breast density category C, is below the mean. As mentioned previously, the tumor for this patient is near the chest wall, which was not scanned in the breast volume using the microwave system. For the patients with breast density category B in Figure 12a, wide variation can be noticed. Two of the patients (10 and 14) have a ratio greater than the average, while patients 1 and 3 are below the average, and patients 11 and 12 have similar average permittivities for both breasts. Among the 14 patients, only patient 7 has a fatty breast (density category A). The ratio for the CC position is higher than the average of healthy volunteers but lower than most of other breast categories. However, as mentioned before, this patient presents with multiple lesions, as shown in the first row of Figure 8.

As shown in Figure 12c, the average permittivity ratio for the MLO position is lower than the CC position. This can be attributed to the fact that, in the MLO position, we typically have a smaller portion of the breast within the microwave system compared to the CC position. This can be observed in the last image of Figure 9.

Two‐way ANOVA was conducted for both healthy participants and cancer patients, using views (LCC, RCC, LMLO, RMLO) and participant groups (cancer, healthy) as the main effects, along with their interaction. Average permittivity, separation distance and breast area were analyzed. Participant groups showed statistically significant results for all measures, whereas views and interaction effect did not show statistically significant results. However, the healthy participant group included younger women who may be expected to have greater breast density and hence permittivity values, resulting in greater average properties and range of properties for this group (see Tables 1 and 2, as well as Figures 3 and 9). Next, the ratio of the average permittivity was analyzed with one‐way ANOVA. For both healthy participants and cancer patients, the CC and MLO views did not exhibit a significant difference (healthy participants: *t*‐value = 1.1, *p*‐value = 0.2; cancer patients: *t*‐value = 1.6, *p*‐value = 0.2). We also compared the ratio of the average permittivity between healthy and cancer participants for CC and MLO views, respectively. This showed statistically significant results for CC views (*t*‐value = 12, *p*‐value = 0.001), while MLO views were not statistically significant (*t*‐value = 0.02, *p*‐value = 0.9). This is likely due to challenges in detecting the tumor in the MLO views, as described above, but does illustrate the potential for tumor detection via comparison of the CC views.

## 4 CONCLUSION

In this paper, a MW imaging system was used to collect scans of both breasts of healthy participants and cancer patients in CC and MLO views. The scans of healthy participants provided insight into the expected similarity between right and left breasts, as well as between CC and MLO views. Statistical analysis did not identify differences between the views when considering average permittivity, separation distance or area. The scans of the cancer patients demonstrated the utility of collecting both CC and MLO views to maximize visibility of the tumor. The ability to detect tumors by comparing the reconstructed permittivity of both breasts was explored, considering both average properties and segmented images. The range of permittivity values, likely resulting from differences in breast composition, resulted in lack of statistically significant differences between the breasts with and without the tumor. However, the ratio of the average permittivity for the CC view was statistically significant when comparing the cancer patients with healthy volunteers. Larger patient studies are required to confirm these findings and the data collected in this study may be used to calculate sample sizes for these investigations.

We note that the patients in this study were scheduled for neoadjuvant chemotherapy and the initial microwave scans of these patients were analyzed to examine the feasibility of detecting tumors with the TMS. This is an important step in validating the technology. In future work, the serial scans collected for each patient during treatment will be analyzed and reported. This is an important next‐step in understanding the potential of this technology for treatment monitoring. Because the TMS is portable, compact, and lightweight, it is suitable for use in rural or remote areas that are under served by health care. However, careful validation of the technology is required, including scanning patients before biopsy.

MW imaging has been previously used to explore detection of breast tumors. The majority of systems are radar‐based, providing localized responses that must be interpreted for detection. Tomography has been used to map the MW properties, enabling detection by identifying increases in properties. This is the first paper to report collection of CC and MLO views of the breast with a MW imaging system. Comparison with scans of healthy participants provides context for exploring tumor detection. The focus of this paper is on average properties, but segmentation shows potential for improved detection.

## CONFLICT OF INTEREST STATEMENT

Elise Fear and Jeremie Bourqui are co‐founders of Wave View Imaging.
